# Bone diseases and the COVID-19 pandemic

**DOI:** 10.20945/2359-3997000000548

**Published:** 2022-11-11

**Authors:** Francisco Bandeira, John P. Bilezikian

**Affiliations:** 1 Universidade de Pernambuco Faculdade de Ciências Médicas Hospital Agamenon Magalhães Recife PE Brasil Divisão de Endocrinologia e Diabetes, Hospital Agamenon Magalhães, Faculdade de Ciências Médicas, Universidade de Pernambuco, Recife, PE, Brasil; 2 Columbia University Vagelos College of Physicians and Surgeons Endocrinology Division New York NY USA Department of Medicine, Endocrinology Division, Vagelos College of Physicians and Surgeons, Columbia University, New York, NY, USA

Although much has been learned about the acute consequences of SARS-CoV-2 infection, the chronic impact on human health is still largely unknown (1). In addition to respiratory symptoms, general features such as fatigue, myalgias and joint pain may persist, usually associated with low-grade inflammation (1). Acute SARS-CoV-2 infection in its moderate to severe form can lead to a high mortality rate especially in vulnerable individuals such as the elderly, a subgroup of patients in whom common muscle-skeletal disorders frequently occur. Crosstalk between skeletal muscle and bone is modified by the ageing process with increased myostatin secretion by myocytes, as well as sclerostin by osteocytes, and changes in the RANK/osteoprotegerin ratio (2).

In the acute phase of SARS-CoV-2 infection, the muscle-skeletal consequences vary according to the severity of infection, including the need of hospitalization due to respiratory failure (3). This may cause delays or interruptions in osteoporosis treatment, which may ultimately result in a fracture and other co-morbidities.

SARS-CoV-2 infection causes a systemic inflammatory response often referred to as cytokine storm (4). This occurs due to the virus entering the cells following ACE2 receptor binding with the induction of an inflammatory cascade, mediated by cytokine secretion from T helper 1 cells and neutrophilic extracellular traps (NETs) (4). The cascade of systemic inflammation may have deleterious effects on skeletal muscle mass and may also lead to increased bone turnover with the consequent bone loss and sarcopenia among survivors (2,5,6). In one trial, bone mineral density decreased due to SARS-CoV-2 infection (7). Volumetric bone mineral density (BMD) by computed tomography rapidly was reduced during the 3 months following an acute infection by more than 8% (7).

Prolonged duration of respiratory failure with oxygen therapy by nasal catheter or intubation, followed by mechanical ventilation, may lead to deficient nutrition (8). This may also have a great impact on the muscle-skeletal system (8,9).

The lockdown phase, which caused a substantial decrease in physical activity, also had a profound effect on muscle-skeletal system (9). This may also decrease sun exposure leading to vitamin D deficiency (6,10,11).

During 2020, the first year of SARS-CoV-2, there was a 25% to 30% increase in the rate of depressive and general anxiety disorders globally (12), which usually require the use of specific pharmacotherapy. The antidepressants may have negative effects on bone (13), in particular the selective serotonin reuptake inhibitors (SSRIs), that can lead to reductions in BMD by dual X-ray absorptiometry (DXA), decreases in volumetric cortical BMD by high-resolution peripheral quantitative computed tomography (HR-pQCT) and impairment in hand grip strength (13).

The treatment of osteoporosis and primary hyperparathyroidism were also affected by the pandemic (14,15) leading to an increase in fracture burden (15). Long-term osteoporosis treatment and monitoring is essential (16). Medications such as bisphosphonates, denosumab and teriparatide do not affect the course of SARS-CoV-2 infection (17). The use of telemedicine increased (18), but the number of surgeries for primary hyperparathyroidism (PHPT) decreased and medical treatment with calcimimetics disproportionately increased (14).

Finally, the diagnosis and management of metabolic bone diseases, such as osteoporosis, has been compromised during COVID-19 pandemic. Guidelines to minimize this impact have been proposed with recommendations for clinicians and patients to avoid delay in starting treatment or inadvertent withdrawal (3). This will ultimately reduce the negative consequences on the skeleton.

In this issue of the AE&M you will find a series of articles addressing an update on a variety of conditions related to calcium and bone metabolism, commonly seen in clinical practice, with new data regarding the effects of SARS-CoV-2 infection as well.
